# Risk Environments, Race/Ethnicity, and HIV Status in a Large Sample of People Who Inject Drugs in the United States

**DOI:** 10.1371/journal.pone.0150410

**Published:** 2016-03-14

**Authors:** Hannah L. F. Cooper, Sabriya Linton, Mary E. Kelley, Zev Ross, Mary E. Wolfe, Yen-Tyng Chen, Maria Zlotorzynska, Josalin Hunter-Jones, Samuel R. Friedman, Don C. Des Jarlais, Barbara Tempalski, Elizabeth DiNenno, Dita Broz, Cyprian Wejnert, Gabriela Paz-Bailey, National HIV Behavioral Surveillance Study Group

**Affiliations:** 1 Rollins School of Public Health at Emory University, 1518 Clifton Road NE, Atlanta, GA 30322, United States of America; 2 ZevRoss SpatialAnalysis, 120 N Aurora St, Suite 3A, Ithaca, NY 14850, United States of America; 3 Institute for Infectious Disease Research, National Development and Research Institutes, 71 West 23^rd^ Street, 4^th^ Fl, New York, NY 10010, United States of America; 4 The Baron Edmond de Rothschild Chemical Dependency Institute, Mount Sinai Beth Israel, 39 Broadway, 5^th^ floor, New York, NY 10006, United States of America; 5 Centers for Disease Control and Prevention, 1600 Clifton Rd, NE (MS-E46), Atlanta, GA 30333, United States of America; National Center for AIDS/STD Control and Prevention, China CDC, CHINA

## Abstract

**Introduction:**

We analyzed relationships between place characteristics and being HIV-negative among black, Latino, and white people who inject drugs (PWID) in the US.

**Methods:**

Data on PWID (N = 9077) were from the Centers for Disease Control and Prevention’s 2009 National HIV Behavioral Surveillance. Administrative data were analyzed to describe the 968 ZIP codes, 51 counties, and 19 metropolitan statistical areas (MSAs) where they lived. Multilevel multivariable models examined relationships between place characteristics and HIV status. Exploratory population attributable risk percents (e-PAR%s) were estimated.

**Results:**

Black and Latino PWID were more likely to be HIV-negative if they lived in less economically disadvantaged counties, or in MSAs with less criminal-justice activity (i.e., lower drug-related arrest rates, lower policing/corrections expenditures). Latino PWID were more likely to be HIV-negative in MSAs with more Latino isolation, less black isolation, and less violent crime. E-PAR%s attributed 8–19% of HIV cases among black PWID and 1–15% of cases among Latino PWID to place characteristics.

**Discussion:**

Evaluations of structural interventions to improve economic conditions and reduce drug-related criminal justice activity may show evidence that they protect black and Latino PWID from HIV infection.

## Introduction

HIV epidemics are heterogeneous across populations and places [1,2]. In the United States (US) in 2011, estimated rates of newly diagnosed HIV cases among people who inject drugs (PWID) were eleven times as high among black PWID (230/100,000), and six times as high among Latino PWID (121/100,000), as among white PWID (21/100,000) [1]. The Centers for Disease Control and Prevention (CDC) and the National HIV/AIDS Strategy prioritized eliminating racial/ethnic disparities in HIV incidence among PWID and other key populations, and highlighted the role of place characteristics (a term used interchangeably here with “environmental features”) in creating disparities [3,4]. To illustrate, PWID are more vulnerable to HIV when they are exposed to environments with more economic disadvantage, drug-related criminal justice activities, and violent crime and other physical and social disorder (e.g., abandoned housing) [5–11], and are less vulnerable to HIV when they live in environments with laws permitting access to sterile syringes without a prescription and with more healthcare services [6,12–15].

HIV-related outcomes, moreover, are heterogeneous *within* racial/ethnic groups of PWID [16,17]. Most notably, these outcomes vary across geographic areas among black, white, and Latino PWID [17,18]. Few studies, though, have investigated place-based determinants of variations in HIV-related outcomes within specific racial/ethnic groups of PWID. Advancing this line of research can help develop structural interventions to address the particular constellation of place characteristics shaping vulnerability and resilience to HIV within each racial/ethnic group.

We cross-sectionally analyzed relationships between features of the environments where PWID live and the odds of being HIV-negative, and explored whether these relationships varied across racial/ethnic groups. Because we analyzed a large (N = 9077) sample of PWID in 19 metropolitan statistical areas (MSAs), we had the rare opportunity to study place-based correlates of HIV within each of three geographic scales: ZIP codes, counties, and MSAs. Our outcome is the odds of being HIV-*negative* because these MSAs had high AIDS prevalences at the time of data collection; being HIV-negative in this context would require ongoing engagement in low-risk behaviors or participation in low-risk networks. The analysis is guided by the Risk Environment Model, a multilevel conceptual framework that highlights the roles that contextual factors play in creating vulnerability and resilience to HIV transmission among PWID [19–23].

We also estimated exploratory racial/ethnic-specific population attributable risk percents (e-PAR%s) for place-based exposures. A previous analysis found large differences in characteristics of the places PWID lived, across and within racial/ethnic groups [24]. By combining data on racial/ethnic-specific exposure to place characteristics with data on the magnitudes of these exposures’ relationships to HIV status, racial/ethnic-specific e-PAR%s estimate the percent of cases of HIV that could potentially have been prevented within each racial/ethnic group if exposure to harmful place-based characteristics were minimized [25,26].

## Materials and Methods

### Study description and analytic sample

We combined 2009 National HIV Behavioral Surveillance (NHBS) data about PWID in 19 US MSAs with data from existing administrative sources to describe the places where PWID lived. NHBS assesses HIV status, HIV-related behaviors, and service use in high-risk populations, including PWID [27].

In 2009, NHBS recruited PWID living in 20 MSAs with the highest AIDS burden in 2006 [18,28,29]. San Juan-Bayamon was excluded from this analysis because the sample was ethnically homogenous (98% were Latino). NHBS used respondent-driven sampling (RDS) to recruit approximately 500 adult (≥18 years old) PWID in each MSA [18]. In total, 9,884 PWID were enrolled across the 19 MSAs. Our analytic sample was 9077: we excluded participants who had an incomplete survey; lacked racial/ethnic information or a valid HIV test or ZIP code; or (because of small numbers) identified as transgender or non-Hispanic race other than white or black (alone or in combination).

### Measures

#### HIV status

NHBS offered anonymous HIV testing.[28] Participants with nonreactive screening test results were considered HIV-negative. Participants were considered HIV-positive if their screening test was reactive and confirmed by Western blot or immunofluorescence assay.

#### Individual race/ethnicity

As described elsewhere [16,24], we used self-report data to construct three mutually exclusive racial/ethnic groups: Latino, non-Hispanic white, and non-Hispanic black (hereafter referred to as white and black, respectively).

#### Geographic areas

Participants reported the ZIP code and county where they lived. Homeless participants’ ZIP codes and counties were based on where they usually slept. Participants were linked to MSAs via data collection site.

#### Other individual-level covariates

Information about participant sociodemographic characteristics, years since first injection, and injection frequency were drawn from NHBS.

#### Place characteristics

We measured features of PWID’s environments in 2009 in ZIP codes, counties, and/or MSAs in four domains: social, economic, healthcare service/criminal justice intervention, and physical environment (Table 1). Methods used to create these measures are described elsewhere [24].

**Table 1:** Place-based Constructs, Variables, and Data Sources.

Environmental Domain	Construct	Variables (Geographic Scale)	Data Source(s)
**Healthcare services and law enforcement intervention environment**	Exposure to law enforcement	Jail incarceration rate, per 1000 adults, by race/ethnicity (18–64 yrs; Metropolitan statistical area [MSA])	Numerator: number of jail inmates from the 2010 Decennial census. Denominator: number of adults 18–64 yrs, from the 2010 Decennial Census
		Arrest rate for hard drug possession^1^, per 1000 adults (18–64 yrs; county, MSA)	Numerator: Number of drug possession arrests in 2009 from the ICPSR county-level detailed arrest and offense database; Denominator: number of adults 18–64 yrs from the ACS 5-year Estimates (2007–2011)
	Health and law enforcement expenditures	Per capita expenditures on police (MSA)	Numerator: expenditures from the 2007 Census of Governments County Area Finances File. Denominator: total population, from the US Census Bureau Population Estimates Program
		Per capita expenditures on corrections (MSA)	
		Per capita expenditures on health (MSA)	
	Poor access to general health care	Percent of adults (18–64 yrs) who are uninsured (county)	2012–2013 Area Health Resource File^2^
		Percent of residents living in a medically underserved area (county)	2013 Health Professional Shortage Area Dataset^3^
	Spatial access to drug- and HIV-related programs	Spatial access to HIV testing sites (ZIP)^4^	CDC’s 2009 National HIV Prevention Program Monitoring & Evaluation database
		Spatial access to substance abuse treatment programs, (a) overall and (b) specifically to methadone maintenance programs (ZIP)	Calculated using gravity based methods, as described in detail elsewhere [24]. Data on facility site location and type were drawn from the *National Directory of Drug and Alcohol Abuse Treatment Programs* [30].
		Spatial access to syringe exchange programs (SEP)	Calculated using gravity based methods, as described in detail elsewhere [24]. Data on SEP site locations were drawn from Des Jarlais’ 2009 “Dave Purchase Memorial Syringe Exchange Program Survey” [31].
**Social environment**	Availability of sex partners	Male: female sex ratio for adults (18–64 yrs); ZIP, county, MSA)^5^	2010 Decennial Census
	Exposure to violence	Rate of reported violent crimes, per 1000 residents (county, MSA)	Numerator: 2009 reported number of violent crimes, as defined by the FBI, drawn from the *Inter-university Consortium for Political and Social Research (ICPSR) c*ounty-level detailed arrest and offense data. Denominator: population size, drawn from the ACS 5-year Estimates (2007–2011)
	Racial/ethnic composition	Percent of total population who are non-Hispanic white, non-Hispanic black/ African-American, or Latino (ZIP)	American Community Survey (ACS) 5-year Estimates (2007–2011)
	Racial/ethnic residential segregation	Black Isolation Index (MSA)^6^	2010 US Decennial Census
		Latino Isolation Index (MSA)	2010 US Decennial Census
**Economic environment**	Income inequality	Gini Coefficient of Income Inequality (MSA)^7^	2010 Decennial Census
	Exposure to economic disadvantage	Median household income (ZIP; county; MSA)	ACS 5-year Estimates (2007–2011)
		Percent of households below federal poverty line (ZIP; county; MSA)	
		Percent of adults (≥16 yrs) in labor force who are unemployed (ZIP; county; MSA)	
		Percent of adults (≥25 yrs) without a high school diploma or general equivalency diploma (ZIP; county; MSA)	
**Physical environment**	Exposure to abandoned buildings	Density per square mile of abandoned property, (a) overall, and of (b) residential units, and (c) commercial properties (ZIP)	Numerator: number of abandoned housing or commercial properties, from the 2009 United States Postal Service Delivery Statistics Product. Denominator: number of square miles, from the US Census Tiger Files
	Access to alcohol	Density per square mile of businesses licensed to sell alcohol for off-premises consumption (ZIP)	Numerator: number of premises, from 2009 U.S Census Bureau’s Zip Code Business Patterns Denominator: square miles, from US Census Tiger Files

1 “Hard” drugs included opium, cocaine, cocaine derivatives (e.g., crack) and “truly addicting” synthetic or other dangerous non-narcotic drugs.

2 This database contained historical data and so it was possible to capture conditions for 2009.

3 The US Health Resources and Services Administration calculates medically underserved areas using a weighted combination of data on (1) the ratio of primary care physicians to residents; (2) rates of poverty and infant mortality; and the percentage of residents aged ≥65 years. Additional information can be found at http://www.hrsa.gov/shortage/mua/.

4 When data from the Census Bureau were used to calculate ZIP-code level variables, ZIP code tabulation areas were used instead of ZIP codes. ZIP code tabulation areas are Census approximations of ZIP codes.

5 People who were institutionalized (e.g., incarcerated) were excluded from calculations.

6 The isolation index measures “the extent to which minority members are exposed only to one another” within census tracts in an MSA (Massey and Denton, p. 288) and was calculated per Massey and Denton (1988). The isolation index varies from 0 (no isolation) to 100 (complete isolation). A value of 44 for black isolation in an MSA would mean, for example, that there is a probability of 0.44 that the next person that a black resident of the MSA will see in his/her census tract will also be black.

7 The Gini Coefficient ranges from 0.0 (a situation of total equality in which all income generated by a population is equally distributed across all families or households in that population) and 1.0 (a situation of total inequality in which all income generated by a population is held by a single family or household). For more information, see http://www.census.gov/prod/2000pubs/p60-204.pdf. The Gini coefficient generated by the 2010 Decennial Census measures inequality in 2009 income. For this measure alone, we drew MSA-level data directly from the Census Bureau. Note that the Census Bureau’s definitions of MSAs and MSA Divisions included more counties than did those of NHBS. Specifically, the census-delineated MSAs included 19 more counties than the NHBS-delineated MSAs.

Features were selected based on past research about place-based exposures and HIV-related outcomes among PWID and other high-risk populations. The geographic scale at which we operationalized each feature was determined by our conceptualization of the feature itself and data availability. For example, we measured black and Latino isolation (forms of racial/ethnic segregation[32]) within MSAs because segregation develops, in part, when white residents live in the suburbs and work in central cities.[33] Likewise, alcohol outlet density was measured within ZIP codes because it is a local phenomenon with local effects. While violent crime, incarceration, and arrest rates may be salient within ZIP codes,[34–41] ZIP-level data on these constructs was unavailable across the 19 MSAs, and so these constructs were assessed in counties and MSAs.

### Analysis

Because measures of place characteristics were often correlated, we used principal components analysis (PCA) with varimax rotation to reduce potential multicollinearity in multivariable models. PCAs combine correlated variables to form uncorrelated components [42]. PCAs were conducted within each geographic scale and domain, and components created for subsets of correlated variables; resulting component scores were standardized (Table 2).

**Table 2:** Components Generated by the Principal Components Analysis.

Component Name (Geographic Scale) and Constituent Variables	Correlations of constituent variables with the component	Values of constituent variables at…	
		…1 SD below the component mean	… 1 SD above the component mean
**HEALTHCARE SERVICE AND LAW ENFORCEMENT INTERVENTION ENVIRONMENT**			
Poor access to general healthcare (county)^1^			
Percent of residents who are uninsured	0.81	19.28	34.15
Percent of residents living in a medically underserved area	0.81	7.14	54.82
Criminal justice (MSA)^2^			
Expenditures on policing per capita	0.87	300.84	393.65
Expenditures on corrections per capita	0.77	67.98	142.40
Hard drug arrest rates, per 1000 adults	0.60	3.33	5.94
**SOCIAL ENVIRONMENT**			
Social component (MSA) ^3^			
Violent crime rate per 1000	0.77	4.89	8.30
Black isolation	0.80	35.08	62.64
Latino isolation	-0.39	33.55	14.67
**ECONOMIC** **ENVIRONMENT**			
Economic disadvantage component (MSA)^4^			
Median income	-0.91	$70609.34	$47369.67
Percent in poverty	0.97	12.05	18.95
Percent unemployed	0.77	8.12	12.36
Percent of adults without a high-school degree/GED	0.77	13.30	20.96
Economic disadvantage component (county)^5^			
Median income	-0.96	66822.19	40327.56
Percent in poverty	0.95	11.75	22.00
Percent unemployed	0.84	8.10	12.61
Percent of adults without a high-school degree/GED	0.86	13.19	20.87
Economic disadvantage component (ZIP) ^6^			
Median income	-0.92	59722.56	28190.61
Percent in poverty	0.93	14.95	35.54
Percent unemployed	0.78	8.97	17.22
Percent of adults without a high-school degree/GED	0.80	15.54	35.28
**PHYSICAL ENVIRONMENT**			
Physical disorder (ZIP) ^7^			
Density of abandoned businesses	0.85	28.25	466.58
Density of businesses licensed to sell alcohol for off-premises consumption	0.85	6.05	78.14

1 The eigenvalue for this component was 1.31 and it accounted for 65.58% of the variance. Eigenvalues describe the variance accounted for by the component.

2 The eigenvalue for this component was 1.72 and it accounted for 57.15% of the variance.

3 The eigenvalue for this component was 1.38 and it accounted for 45.88% of the variance.

4 The eigenvalue for this component was 2.97 and it accounted for 74.28% of the variance.

5 The eigenvalue for this component was 3.25 and it accounted for 81.32% of the variance.

6 The eigenvalue for this component was 1.44 and it accounted for 72.05% of the variance.

7 The eigenvalue for this component was 2.95 and it accounted for 73.86% of the variance.

We used descriptive statistics to characterize the sample and the places where PWID lived. Racial/ethnic-specific coefficients of variation were calculated for place-based exposures to quantify dispersion around the mean within racial/ethnic groups. Model building occurred in four stages:

#### Stage 1

Bivariate hierarchical generalized linear models (HGLMs) were constructed to examine the relationship of each feature (whether a PCA-derived component or a single variable) to the odds of being HIV-negative, and to determine whether individual race/ethnicity moderated this association. (We use the term “bivariate” here to describe models that include a single place-based covariate, indicator variables for individual race/ethnicity, and the interactions of the place-based covariate with these indicator variables.). In all HGLMs, four-level models were constructed (individuals nested in ZIPs; ZIPs in counties; and counties in MSAs) that included random intercepts for each scale. Features associated with the outcome at p<0.05 (as main effects or interacted with race/ethnicity) were carried forward into Stage 2. The race by variable interactions were coded with whites as the reference group, thus the p-values and estimates for blacks and Latinos represent ratios of odds ratios (ORs) and the significance of the difference in ORs compared to whites. To facilitate interpretation, we also calculated some racial/ethnic-specific ORs using model estimates (presented in text). The racial/ethnic-specific estimate was calculated by multiplying the ratio by the OR in whites: OR black/OR white * OR white = OR black.

#### Stage 2

To further reduce the number of place-based variables analyzed within each geographic scale, we created three four-level multivariable HGLMs, one for each geographic scale, and used backward selection to eliminate variables (p<0.05 cutpoint). Backward selection was used because the Risk Environment Model does not specify *which* variables to include in each domain.

#### Stage 3

A multivariable HGLM was constructed containing all significant environmental features (within ZIP codes, counties, and MSAs) from Stage 2, individual-level race/ethnicity, and possible individual-level confounders (e.g., age, gender). Backward selection (p<0.05 cutpoint) was used to make the model more parsimonious. We re-ran this multivariable model with select possible individual-level mediators of relationships between place-based exposures and HIV status (e.g., income, injection frequency) to learn whether individual characteristics mediate relationships.

#### Stage 4

We estimated racial/ethnic-specific PAR%s for each place-based exposure that was significantly associated with the outcome in the model that controlled for age and gender. PAR%s require that the sample represent the underlying population and that the exposure cause the outcome [26]. Because we can make neither claim, we call these PAR%s “exploratory PAR%s” (e-PAR%s) and did not calculate them when we suspected reverse causation. All analyses were run on Stata version 13.

### Ethics

The Emory University Institutional Review Board (IRB) approved study protocols. All state and local jurisdictions participating in NHBS obtained human subject protections approval before conducting the 2009 NHBS survey among PWID. Activities for NHBS were approved by local IRBs for each of the 20 participating cities and by the CDC as research in which the CDC was not engaged.[43–45] All participants provided verbal informed consent to take part in the interview and to be tested for HIV. Verbal rather than written consent procedures were used to protect participants. Verbal consent was documented electronically on the survey instrument by interviewers for all participants and on hard copy as required by local IRBs. All consent procedures, including verbal consent, were approved by local IRBs

## Results

The 9077 PWID lived in 19 MSAs, 51 counties, and 968 ZIP codes. Approximately half (51.6%) were black, 30.3% were white, and 18.1% were Latino (Table 3). Most (71.1%) were men and average age was 45.7 (standard deviation [SD] = 10.6). Participants were impoverished: 60.9% subsisted on <$10,000 a year and 39.8% were homeless. Approximately 9% tested positive for HIV. Prevalence varied by race/ethnicity: 10.7% of black PWID tested positive, as did 7.6% of Latino PWID, and 6.3% of white PWID.

**Table 3:** Characteristics of the sample of people who inject drugs (PWID), drawn from the 2009 Centers for Disease Control and Prevention’s National HIV Behavior Surveillance.

Characteristic	No. (%) or mean (SD)
	N = 9077
**Age (yrs)**	45.7 (10.6)
**Sex**	
Male	6504 (71.7%)
Female	2573 (28.4%)
**Race/ethnicity**	
Non-Hispanic Black/African-American	4687 (51.6%)
Non-Hispanic White	2750 (30.3%)
Latino/Latino	1640 (18.1%)
**Annual household income (USD)**	
<$10,000	5503 (60.9%)
$10,000- $19,999	2082 (23.1%)
≥$20,000	1446 (16.1%)
**High-school graduate/General equivalency diploma**	6043 (66.6%)
**Employed full-time**	400 (4.4%)
**Currently homeless**	3649 (39.8%)
**Injection Frequency**	
Daily	6729 (74.3%)
Less than daily	2329 (25.7%)
**Number of years since first injection**	23.3 (13.0)
**Tested positive for HIV**	
Overall	799 (8.8%)
Black participants	504 (10.7%)
Latino participants	125 (7.6%)
White participants	172 (6.3%)

As reported elsewhere [24], across most measures of place-based exposures, black PWID lived in more disadvantaged areas than white and (in most cases) Latino PWID (Table 4). Coefficients of variation reveal moderate (25%-75%) to high (>75%) variation in exposure to place characteristics within each racial/ethnic group.

**Table 4:** Characteristics of the ZIP Codes (N = 968), Counties (N = 51), and Metropolitan Statistical Areas (MSAs; N = 19) where the 9,077 participants of the 2009 Centers for Disease Control and Prevention’s National HIV Behavioral Surveillance Lived, by Racial/Ethnic Group^1^.

Feature and geographic scale	White		Black		Latino		Coefficient of Variation		
	Mean or %	SD or N	Mean or %	SD or N	Mean or %	SD or N	White	Black	Latino
**Healthcare service and law enforcement intervention environment**									
*Criminal-Justice Component (MSA)*	0.02	1.06	-0.17	0.88	0.34	0.95	6015.24	-514.28	282.36
Healthcare expenditures, per capita (USD; MSA)	172	176	135	139	150	147	102.33	102.96	98.00
Incarceration rate, per 1000 (MSA)	0.33	0.16	0.33	0.17	0.29	0.11	0.48	0.52	0.38
Hard drug possession arrest rate, per 1000 (county)	5.16	4.43	6.24	6.65	5.73	2.19	85.85	106.57	38.22
*Poor access to general healthcare component (county)*	0.10	1.02	0.39	0.93	0.26	0.64	10.2	2.38	2.46
≥1 HIV testing sites (ZIP)	74.7%	2053	86.7%	4063	82.9%	1360	N/A	N/A	N/A
≥1 drug treatment facility (ZIP)	93.3%	2567	93.3%	4374	93.5%	1534	N/A	N/A	N/A
≥1 methadone maintenance program (ZIP)	69.9%	1922	72.9%	3419	65.5%	1074	N/A	N/A	N/A
≥1 syringe exchange program (ZIP)	46.1%	1267	42.4%	1986	50.2%	823	N/A	N/A	N/A
**Social Environment**									
*Social Component (MSA)*	-0.24	0.84	0.28	1.02	-0.30	0.62	350.00	364.29	206.67
Male: female sex ratios									
ZIP	1.12	0.39	1.00	0.26	1.05	0.24	34.82	26.00	22.86
County	0.97	0.06	0.95	0.05	0.96	0.06	6.19	5.26	6.25
MSA	0.97	0.04	0.96	0.03	0.96	0.03	4.12	3.13	3.13
Violent crime rate (per 1000; county)	7.31	3.15	9.14	3.60	6.93	2.20	43.09	39.39	31.75
Racial/ethnic composition (ZIP)									
% white	43.60	23.87	19.10	19.29	25.13	22.88	54.75	100.99	91.05
% black	20.51	21.67	56.50	30.01	22.76	21.74	105.66	53.12	95.52
% Latino	22.77	19.94	18.01	20.47	43.14	24.80	87.57	113.66	57.49
**Economic environment**									
*Economic Disadvantage Component*									
MSA	-0.11	0.76	0.07	1.04	0.25	0.76	6.91	14.86	3.04
County	0.41	0.84	0.71	0.75	0.73	0.74	204.63	105.58	101.48
ZIP	0.24	0.94	0.87	0.87	0.69	0.92	3.92	1.00	1.33
Gini coefficient of income inequality (MSA)	47.29	2.10	46.66	1.97	48.26	2.14	4.44	4.22	4.43
**Physical environment**									
*Physical disorder component*	0.28	1.13	-0.09	0.50	0.25	0.93	4.04	5.56	3.72
Density of abandoned residential properties per sq mile (ZIP)	83.4	107.21	175.79	207.19	93.08	108.19	128.55	117.86	116.23

1 Components generated through the principal components analysis are *italicized*.

Findings are discussed by domain, below. Overall, multivariable results indicated that several place-based exposures were related to the odds of being HIV-negative among black and Latino PWID, while few were related to HIV status among white PWID.

### Healthcare Service/Criminal Justice Intervention Environment

Bivariate and multivariable models, which controlled for significant participant characteristics, suggested an association between the criminal justice component and HIV status in black (p = 0.001) and Latino PWID (p = 0.04) that was different from whites (Table 5; Table 6; Table 7, Model B). More specifically, black PWID were less likely to be HIV-negative if they lived in MSAs that scored higher on the criminal-justice component than if they lived elsewhere (race-specific OR = 0.68; adjusted OR [AOR] = 0.64); multivariable analyses suggest the same for Latino PWID (AOR = 0.65). Specifically, multivariable models indicate that black and Latino PWID were about 35% less likely to be HIV-negative if they lived in an MSA that was 1 SD above the mean on the criminal-justice component. MSAs that were 1 SD above the mean on this component spent $92.8 per capita more on policing, $74.4 per capita more on corrections, and had drug-related arrest rates that were 2.6/1000 higher than the mean MSA. In MSAs ≥1 SD above the mean on this component, e-PAR%s suggest that 8.35% of HIV infections among black PWID possibly might have been prevented if drug-related arrest rates and spending on corrections and policing were at their mean values for MSAs in the sample, as might 15.23% of infections among Latino PWID.

**Table 5:** Bivariate associations between HIV negative status and (a) features of the environments where people who inject drugs (N = 9,077) lived when participating in the 2009 National HIV Behavioral Surveillance, and (b) participant characteristics^1^.

Place-based characteristics												
Feature (geographic scale)	Models with place characteristic only		Models including interactions of place characteristics with individual race/ethnicity									
			Effect of place characteristic among white participants (ref)		Effect of place characteristic among black participants (ref = white)		Effect of place characteristic among Latino participants (ref = white)		Main effect of black race (ref = white)		Main effect of Latino ethnicity (ref = white)	
	OR	p-value	OR	p-value	OR	p-value	OR	p-value	OR	p-value	OR	p-value
**HEALTHCARE SERVICE AND LAW ENFORCEMENT INTERVENTION ENVIRONMENT**												
**MSA**												
*Criminal-justice component*^2^	0.79	0.16	0.85	0.36	0.80	0.02	1.04	0.79	0.56	<0.0005	0.85	0.27
Healthcare expenditures per capita	1.00	0.57	1.00	0.34	1.00	0.3	1.00	0.60	0.49	<0.0005	0.82	0.28
Incarceration rate per 1000	0.49	0.49	1.04	0.98	0.60	0.43	0.06	0.004	0.63	0.06	2.04	0.03
**County**												
Hard drug possession arrest rate per 1000	0.99	0.68	0.99	0.83	0.99	0.75	1.10	0.08	0.57	0.001	0.52	0.05
*Poor access to general healthcare component*	1.00	0.99	0.99	0.95	1.01	0.96	1.74	0.003	0.56	<0.0005	0.80	0.11
**ZIP**												
≥1 HIV testing site	0.51	<0.0005	0.56	0.02	0.81	0.54	1.32	0.46	0.67	0.21	0.71	0.32
≥1 drug treatment facility	0.83	0.37	0.86	0.67	1.16	0.69	0.52	0.24	0.47	0.04	1.61	0.36
≥1 methadone maintenance facility	0.69	0.007	0.54	0.006	1.71	0.03	0.86	0.62	0.37	<0.0005	0.95	0.86
≥1 syringe exchange program	0.84	0.22	0.59	0.01	1.96	0.001	0.81	0.45	0.38	<0.0005	1.00	0.99
**SOCIAL ENVIRONMENT**												
**MSA**												
*Social component*	0.87	0.42	1.20	0.37	0.79	0.10	0.39	<0.0005	0.52	<0.0005	0.75	0.05
More women vs. equity	0.44	0.003	0.76	0.41	0.56	0.007	0.44	0.004	0.72	0.03	1.41	0.13
**County**												
Violent crime rate per 1000	0.96	0.29	1.02	0.71	0.95	0.15	0.88	0.02	0.80	0.45	2.39	0.05
Sex ratios												
More women vs. equity	0.63	0.08	1.69	0.08	0.31	<0.0005	0.39	0.002	0.90	0.53	1.33	0.20
More men vs. equity	0.37	0.15	0.40	0.19	1.00	0.99	0.85	0.72	0.90	0.53	1.33	0.20
**ZIP**												
Percent of residents who are white	1.01	0.001	1.00	0.61	1.01	0.24	1.00	0.92	0.48	<0.0005	0.86	0.52
Percent of residents who are black	0.99	< 0.0005	1.00	0.73	1.00	0.48	1.00	0.50	0.63	0.006	0.98	0.91
Percent of residents who are Latino	1.00	0.30	1.00	0.65	1.00	0.42	1.00	0.69	0.60	0.001	0.78	0.28
Sex ratios												
More women vs. equity	0.88	0.36	0.99	0.97	1.09	0.76	1.04	0.92	0.34	<0.0005	0.61	0.04
More men vs. equity	0.88	0.38	0.45	0.001	2.73	<0.0005	2.18	0.02	0.34	<0.0005	0.61	0.04
**ECONOMIC** **ENVIRONMENT**												
**MSA**												
*Economic Disadvantage Component*	1.28	0.14	1.39	0.10	0.87	0.29	1.30	0.11	0.54	<0.0005	0.83	0.16
Gini	0.94	0.38	1.06	0.46	0.80	< 0.0005	0.86	0.03	20000.00	<0.0005	1111.11	0.03
**County**												
*Economic Disadvantage Component*	0.88	0.31	1.18	0.30	0.69	0.005	0.68	0.04	0.64	<0.0005	1.08	0.66
**ZIP**												
*Economic disadvantage component*	0.90	0.09	0.99	0.89	0.98	0.83	0.92	0.55	0.55	<0.0005	0.94	0.70
Percent unemployed	0.99	0.21	1.04	0.06	0.95	0.04	0.94	0.04	0.90	0.71	1.77	0.14
**PHYSICAL ENVIRONMENT**												
**ZIP**												
*Physical disorder component*	1.01	0.90	0.94	0.48	1.31	0.03	0.95	0.63	0.52	<0.0005	0.89	0.41
Density of abandoned residential properties per square mile	1.00	0.02	1.00	0.30	1.00	0.62	1.00	0.20	0.53	<0.0005	1.04	0.84

1 We use the term “bivariate” here to describe models that include a single place-based covariate, indicator variables for individual race/ethnicity, and the interactions of the place-based covariate with these indicator variables. All bivariate models were hierarchical generalized linear models with four levels (individual nested in ZIP code, ZIP code nested in county, and county nested in MSA).

2 Components generated through the principal components analysis are *italicized*.

**Table 6:** Bivariate associations between HIV negative status and participant characteristics^1^.

Race (white is ref) Black	0.47	0.001
Latino	0.60	0.07
Age	0.98	< 0.0005
Gender	0.93	0.37
Income	1.05	0.02
Homeless	1.48	< 0.0005
Employed (full time)	2.61	0.001
High-school graduate	1.22	0.01
Frequency of injection	0.84	< 0.0005
Years since first injection	0.99	< 0.0005

1 All bivariate models were hierarchical generalized linear models with four levels (individual nested in ZIP code, ZIP code nested in county, and county nested in MSA).

**Table 7:** Multivariable hierarchical generalized linear models regressing the odds of HIV negative status on characteristics of the environments where people who inject drugs (N = 9,077) lived when participating in the National HIV Behavioral Surveillance in 2009^1^.

Features	Model A: Geographic-scale specific multivariable model^2^		Model B: Multivariable model excluding possible individual-level mediators		Model C: Multivariable model including possible individual-level mediators	
	OR	p-value	OR	p-values	OR	p-value
**HEALTHCARE SERVICE AND LAW ENFORCEMENT INTERVENTION ENVIRONMENT**						
**MSA **						
*Criminal-justice component*^3^ interacted with individual race/ethnicity						
White (ref)	0.86	0.23	0.90	0.53	0.89	0.46
Black/whites	0.77	0.009	0.71	0.001	0.71	0.002
Latino/whites	0.83	0.20	0.72	0.04	0.73	0.06
**COUNTY**						
*Poor access to general healthcare component* interacted with individual race/ethnicity						
White (ref)	0.93	0.73	1.05	0.79	1.08	0.69
Black/whites	1.20	0.13	1.21	0.19	1.20	0.22
Latino/whites	2.13	<0.0005	2.27	<0.0005	2.22	0.001
**ZIP **						
≥1 HIV testing site	0.52	<0.0005	0.59	0.006	0.58	0.005
≥1 syringe exchange program site	0.61	<0.0005				
**SOCIAL ENVIRONMENT**						
**MSA **						
*Social Component* interacted with individual race/ethnicity						
White (ref)	1.31	0.17	1.12	0.63	1.16	0.56
Black/whites	0.86	0.36	1.04	0.84	0.96	0.68
Latino/whites	0.51	0.02	0.45	0.01	0.44	0.01
More women vs. men interacted with individual race/ethnicity						
White	0.67	0.21				
Black/whites	0.58	0.03				
Latino/whites	0.53	0.07				
**ZIP **						
Percent of residents who are black	0.99	0.002	0.99	0.005	0.99	0.005
Sex ratios						
More women vs equity: Whites (ref)	0.88	0.66	0.88	0.66	0.88	0.65
More women vs equity: Black/whites	1.29	0.39	1.38	0.30	1.40	0.29
More women vs equity: Latino/whites	0.90	0.76	2.16	0.05	2.09	0.06
More men vs equity: White (ref)	0.54	0.01	0.54	0.009	0.54	0.01
More men vs equity: Black/whites	2.53	<0.0005	2.32	0.002	2.26	0.003
More men vs equity: Latino/whites	2.01	0.04	1.65	0.15	1.60	0.19
**ECONOMIC** **ENVIRONMENT**						
**MSA **						
*Economic disadvantage component* interacted with individual race/ethnicity						
White (ref)	1.23	0.19				
Black/whites	1.01	0.97				
Latino/whites	1.50	0.02				
**COUNTY**						
*Economic disadvantage component* interacted with individual race/ethnicity						
White (ref)	1.25	0.31	1.12	0.59	1.07	0.77
Black/whites	0.61	<0.0005	0.67	0.04	0.73	0.12
Latino/whites	0.54	0.001	0.60	0.04	0.64	0.07
**ZIP**						
Percent unemployed	1.06	<0.0005	1.03	0.03	1.03	0.04
**INDIVIDUAL-LEVEL COVARIATES AND MEDIATORS **						
Race (white is ref)						
Black			0.51	0.003	0.46	0.001
Latino			0.60	0.08	0.60	0.08
Age			0.99	0.07	1.01	0.18
Sex			0.87	0.12	0.89	0.20
Annual Income					1.03	0.16
Currently Homeless					1.48	<0.0005
Employed (Full-time)					2.39	0.002
High-school graduate					1.16	0.07
Frequency of injection					0.84	<0.0005
Number of years since first injection					0.99	0.01
Random Effects						
Var (SE) for MSA			0.28 (0.14)		0.29 (0.14)	
Var (SE) for County			0.03 (0.13)		0.01 (0.13)	
Var (SE) for ZIP			0.19 (0.07)		0.17 (0.07)	

1 Models had four levels: individuals nested in ZIP codes, ZIP codes nested in counties, and counties nested in metropolitan statistical areas (MSAs)

2 Model A presents results after backward selection.

3 Components generated through the principal components analysis are *italicized*.

These cross-sectional analyses indicated an association between HIV status and healthcare access in Latino PWID that was different from whites (p < 0.0005). More specifically, Latino PWID living in counties with worse general healthcare access (i.e., higher percentages of residents were uninsured or lived in medically underserved areas) were more likely to be HIV-negative (racial/ethnic-specific OR = 1.72; AOR = 2.38). They also suggest that PWID, regardless of race/ethnicity, living in ZIP codes with ≥1 HIV testing sites were less likely to be HIV-negative (AOR = 0.59, p = 0.006).

### Social Environment

Bivariate and multivariable analyses also indicated that the relationship between the social component and HIV status was different in Latino PWID compared to whites (p = 0.01). The data showed that Latino PWID living in MSAs with lower values on the social component were more likely to be HIV-negative than Latino PWID living elsewhere (racial/ethnic-specific OR = 0.47; AOR = 0.50. Specifically, Latino PWID were 50% more likely to be HIV-negative if they lived in an MSA that was 1 SD below the mean on the social component. In MSAs that were 1 SD below the mean on this component, the crime rate was 3.4/1000 incidents lower, the black isolation index was 27.6 points lower, and the Latino isolation index was 18.8 points higher than in the mean MSA. However, E-PAR%s suggest that only 0.89% of HIV infections among Latino PWID in MSAs scoring ≥1 SD above the mean on this component could have been prevented if rates of violent crime, black isolation, and Latino isolation were at mean levels, given the low prevalence of this characteristic in Latino PWID.

Regardless of individual race/ethnicity, PWID living in ZIP codes with higher percentages of black residents were less likely to be HIV-negative (AOR = 0.99, p = 0.005). Among PWID living in ZIPs above the 75^th^ percentile on this variable (≥64.2% of residents were black), e-PAR%s suggest that 19.11% of infections among black PWID might be attributable to the racial/ethnic segregation of black residents, as might 3.12% of infections among white and Latino PWID.

Relationships between ZIP-level sex ratios and HIV status varied by race/ethnicity. For example, living in a ZIP code with >105 men for every 100 women was associated with higher odds of being HIV-negative among black PWID (racial/ethnic-specific AOR = 1.25), but with *lower* odds of being HIV-negative among white PWID (AOR = 0.54).

### Economic Environment

Bivariate and multivariable models suggest a significant difference in association with economic disadvantage and HIV status in black (p = 0.04) and Latino PWID (p = 0.04) compared to whites. Black and Latino PWID living in counties that scored higher on the economic disadvantage component were less likely to be HIV-negative than black and Latino PWID living elsewhere (black PWID: OR = 0.82; AOR = 0.75; Latino PWID: OR = 0.81; AOR = 0.68). Specifically, black and Latino PWID were 25% and 32% less likely, respectively, to be HIV-negative if they lived in a county that scored 1 SD above the mean on the economic disadvantage component. In counties scoring 1 SD above the mean on this component, the poverty rate was 10.3 percentage points higher, the unemployment rate was 4.5% percentage points higher, and the high-school dropout rate was 7.7 percentage points higher than in the mean county, and the median income was $26,494 lower. In counties that were ≥1 SD above the mean on this component, e-PAR%s suggest that 10.04% of HIV infections among black PWID might have been prevented if these counties’ economic conditions were at the mean, as might 12.62% of infections among Latino PWID.

A one-unit increase in ZIP-code unemployment rates was associated with a 3% increase in the odds of being HIV-negative, regardless of race/ethnicity (AOR = 1.03, p = 0.03).

### Physical Environment

The physical environment was unrelated to the odds of being HIV-negative in all racial/ethnic groups.

Including possible individual-level mediators in the multivariable model did not substantively alter the magnitudes of relationships between place-based exposures and the outcome (i.e., no AOR for place-based exposures differed by ≥10% across Models B and C, Table 7), suggesting that these covariates did not mediate these associations.

## Discussion

In the 19 US MSAs with the highest AIDS burden in 2006, features of social, economic, and healthcare service/criminal justice environments were associated with odds of being HIV-negative among black and Latino PWID, and were rarely associated with this outcome among white PWID. Features of PWID residential environments varied both across and within racial/ethnic groups of PWID [24]. E-PAR%s suggest that percentages of cases attributable to place characteristics were higher for black and Latino PWID than white PWID.

As others have noted [23,46,47], almost all multilevel public health studies of individuals nested in places exclusively focus on exposures operating within neighborhoods (e.g., census tracts). Neighborhoods, however, do not exist in isolation, and factors operating within other geographic scales may also influence health. By analyzing CDC data on 9o77 PWID living in 19 MSAs, we were able to explore characteristics of *multiple* geographic scales simultaneously, and found that features of ZIP codes, counties, and MSAs were related to the odds of being HIV-negative, controlling for characteristics of other geographic scales.

Consistent with past research [5], we found that black and Latino PWID were more likely to be HIV-negative if they lived in MSAs that scored lower on the criminal-justice component–that is, if they lived in MSAs that spent less on police and corrections and had lower drug-related arrest rates; this relationship persisted after controlling for characteristics of individuals, ZIP codes and counties. Risk behaviors and networks may mediate these relationships. PWID living in New York City health districts with lower drug-related arrest rates are less likely to engage in receptive syringe sharing [6,12]. Additionally, PWID’s sexual and injecting networks may experience less turnover when fewer network members are cycling through jail/prison [48,49], and may have lower background HIV seroprevalence [5]. Several jurisdictions have relaxed drug-related laws and their enforcement, or sought to reduce incarceration rates [50]. Longitudinal research should explore whether these changes affect HIV transmission among black and Latino PWID. The persistence of the relationship between the criminal-justice component and being HIV-negative in multivariable models controlling for violent crime suggests that perhaps some MSAs addressed violent crime using community-based strategies or alternatives to incarceration, rather than relying on criminal-justice approaches.

Controlling for possible confounders operating at other geographic scales, we found that black and Latino PWID living in less economically disadvantaged counties were more likely to be HIV-negative, as were Latino PWID living in MSAs with less violent crime and black isolation, and more Latino isolation. Residents of places that are less economically disadvantaged or less violent report less psychological distress [51–57]; better psychological well-being may have protected participants in these areas from engaging in sexual and injecting risk behavior [58–60]. Additionally, black and Latino PWID’s injecting and sexual networks may have lower HIV prevalence in less economically disadvantaged and violent areas [10,11,61–65]. The positive association between ZIP-code unemployment rates and being HIV-negative is likely a measurement artifact: the unemployment rate’s numerator excluded unemployed people who stopped actively seeking work.

Relationships between residential segregation and health are complex. Latino isolation can indicate Latino enclaves, which may provide social resources that promote resilience [66,67], including resilience to HIV transmission. Additionally, recent Latino immigrants are more likely to live in enclaves,[68] and may have lower HIV prevalence [69]. While predominately black neighborhoods in MSAs with high black isolation can also foster resilience [70], they tend to have fewer economic resources and higher rates of violent crime, and may be targeted by aggressive policing [33,71–73], each of which might foster HIV transmission [6,7,10,11,61–65,74]. Additionally, racial/ethnic assortativity in injecting and sexual partnerships among black adults [75,76], combined with historically high HIV prevalence in this population [77,78], increase the chances that black PWID will have HIV-positive partners. Perhaps for these reasons (and because final models did not control for these factors within ZIP codes), the percent of residents who were black in PWID’s ZIP codes was inversely associated with being HIV-negative, regardless of PWID race/ethnicity.

Past cross-sectional NHBS analyses identified substantial geographic variation in HIV status among white PWID [16,79], suggesting that variations in place-based exposures might be associated with HIV infection in this group. Few place characteristics, however, were associated with HIV among white PWID, perhaps because HIV prevalence was relatively low (6.3%).

PAR%s are powerful but underused tools [25,80,81]: by combining information on effect size and exposure prevalence, they estimate the percentage of cases in a population attributable to an exposure, and thus help prioritize intervention targets [25]. PAR%s can help illuminate the role of place in shaping HIV (and other health outcomes) across and within racial/ethnic groups. Black, Latino, and white PWID live in markedly different environments in the US [24]. Racial/ethnic-specific PAR%s incorporate this variation in exposure prevalence, while ORs and other effect estimates ignore it, though we caution that we report *exploratory* racial/ethnic-specific PAR%s because we could not claim causality or a representative sample. E-PAR%s preliminarily suggest that the environmental features studied here account for few HIV cases among whites, 8%-19% of cases among black PWID, and 1%-15% among Latino PWID. They suggest that structural interventions to eliminate HIV incidence among black and Latino PWID might prioritize alleviating economic disadvantage (black e-PAR% = 10%; Latino e-PAR% = 12%), and accelerating the transition from applying criminal-justice approaches to drug activity to public health approaches (black e-PAR% = 8%, Latino e-PAR% = 15%). The percentage of ZIP-code residents who are black likely reflects local socioeconomic deprivation [33,71–73] and the self-perpetuating nature of historically high HIV prevalence among black adults (black e-PAR% = 19%; Latino and white e-PAR% = 3%). Structural interventions should be developed and evaluated to improve socioeconomic conditions and increase access to HIV testing and treatment in predominately black ZIP codes.

We attribute the inverse relationships between access to healthcare services and being HIV-negative to the analyses’ cross-sectional design, and to successful efforts to locate health services in high-need areas (e.g., [82]).

### Limitations

On average, HIV-positive PWID reported being diagnosed 12 years before participating in NHBS. While HIV-positive participants reported living in the same MSA for 33 years, on average, they may have moved to new ZIP codes or counties post-infection. If HIV-positive individuals were able to qualify for services post-diagnosis that moved them to “better” counties or ZIP codes, ORs for county- and ZIP-level exposures may be biased toward the null. If their infection led them to “worse” areas, ORs may be inflated. HIV-positive individuals may have been more or less likely to move than HIV-negative individuals for many reasons, including depression and discrimination; the possible effect of differential relocations on the statistical relationships identified here is unknown.

ZIP code areas are designed to facilitate mail delivery and may not capture PWID activity spaces. The resulting exposure misclassification likely biased ORs to the null.

The NHBS sample may not represent the underlying population of PWID in the 19 MSAs. While characteristics of the true underlying PWID populations are unknown, RDS generates samples different from those generated using other methods [83,84]. Additionally, four-level HGLMs could not adjust for possible clustering of HIV within RDS recruitment chains.

Past HIV prevalence in a population predicts individual serostatus.[85,86] While we were unable to control for racial/ethnic-specific seroprevalence, we re-ran models controlling for MSA-level racial/ethnic-specific AIDS-related mortality rates among PWID and found no differences in the magnitudes of relationships between place characteristics and HIV (data available upon request).

## Conclusions

Features of the social, economic, healthcare service/criminal justice intervention environments in ZIP codes, counties, and MSAs were associated with the odds of being HIV-negative among black and Latino PWID. E-PAR%s suggest that 7%-19% of HIV cases among black PWID and 3%-24% of cases among Latino PWID might be attributable to these features. To help eliminate HIV transmission among black and Latino PWID, structural interventions could be implemented that reduce economic disadvantage, accelerate the transition away from criminal-justice approaches to drug activity to public health approaches, and target mediators of relationships between these place-based exposures and HIV transmission. Evaluating effects of such interventions on being HIV-negative is important for addressing racial disparities in HIV status and for reducing HIV incidence. Focusing these efforts in predominately black ZIP codes may be particularly beneficial for black PWID.
